# Bridging Gaps in Migraine Management: A Comprehensive Review of Conventional Treatments, Natural Supplements, Complementary Therapies, and Lifestyle Modifications

**DOI:** 10.3390/ph18020139

**Published:** 2025-01-22

**Authors:** Fatma Abo-Elghiet, Heba Elosaily, Doha K. Hussein, Riham A. El-Shiekh, Ashraf A’aqoulah, Einas M. Yousef, Heba Mohammed Refat M. Selim, Ahmed M. El-Dessouki

**Affiliations:** 1 Department of Pharmacognosy and Medicinal Plants, Faculty of Pharmacy (Girls), Al-Azhar University, Cairo 11754, Egypt; fatmaaboelghiet731.el@azhar.edu.eg; 2 Biochemistry Department, Faculty of Pharmacy, Ahram Canadian University, 4th Industrial Region, 6th of October City 12585, Egypt; heba.elosaily@acu.edu.eg; 3 Department of Pharmacognosy, Faculty of Pharmacy, Cairo University, Cairo 11562, Egypt; doha.khaled@pharma.cu.edu.eg; 4 Department of Public Health, College of Public Health and Health Informatics, King Saud bin Abdulaziz University for Health Sciences, Riyadh 11481, Saudi Arabia; 5 King Abdullah International Medical Research Center, Riyadh 11481, Saudi Arabia; 6 College of Medicine, Alfaisal University, Riyadh 11533, Saudi Arabia; eyousef@alfaisal.edu; 7 Department of Pharmaceutical Sciences, College of Pharmacy, AlMaarefa University, P.O. Box 71666, Riyadh 11597, Saudi Arabia; hmustafa@um.edu.sa; 8 Department of Pharmacology and Toxicology, Faculty of Pharmacy, Ahram Canadian University, 6th of October City 12566, Egypt; ahmed.desoky@acu.edu.eg

**Keywords:** migraine, complementary therapies, alternative approaches, natural supplements

## Abstract

**Background**: Migraine, a complex neurological condition, poses significant challenges for both sufferers and healthcare providers. While prescription medications play a vital role in managing migraine attacks, the quest for natural, non-pharmacological alternatives has garnered increasing interest. This review explores the efficacy and safety of natural supplements as treatments for migraine relief, comparing them with conventional prescription medications. **Methods**: The review delves into herbal supplements, clinical studies on natural remedies, aromatherapy, dietary influences, and lifestyle modifications in the context of migraine management in several databases. **Results**: The findings shed light on the potential of natural supplements as complementary or alternative approaches to traditional migraine therapies, offering insights into a holistic and personalized treatment paradigm for migraine sufferers. **Conclusions**: Natural supplements have gained attention as potential treatments for migraine relief, often perceived as safer alternatives to conventional medications.

## 1. Introduction

Migraine is a common and debilitating neurological disorder that affects individuals across all age groups. It is a leading cause of disability worldwide, with significant societal and personal impacts, particularly during the most productive years of life [1,2,3,4]. Migraine is characterized by recurrent headaches that are typically unilateral or bilateral, pulsating in nature, and often accompanied by symptoms such as nausea, vomiting, photophobia, and phonophobia [5]. The International Classification of Headache Disorders (ICHD-3) identifies migraine as a primary headache disorder, distinct from more than 250 other types of headaches [6]. Despite its significant impact, the mechanisms underlying migraines remain incompletely understood. Research points to a combination of genetic predisposition, neurovascular processes, and inflammatory pathways, including the activation of the trigeminovascular system and the release of neuropeptides such as calcitonin gene-related peptide (CGRP) [7,8,9,10].

Conventional migraine treatment focuses on acute symptom relief and prevention, with options primarily including triptans and nonsteroidal anti-inflammatory drugs (NSAIDs) for acute management, as well as preventive medications like beta-blockers and CGRP inhibitors [11]. However, limitations such as medication overuse headaches, adverse effects, and contraindications in certain populations highlight the need for alternative and complementary therapies [12]. Several natural products such as ginger, butterbur, turmeric, feverfew, feverfew+ white willow, and ginkgo have promising potential for the management of migraine [13].

This comprehensive review addresses these gaps by synthesizing current knowledge on natural and complementary approaches, including herbal products (plant extracts, pure compounds, or their supplements), dietary supplements (non-herbal agents), aromatherapy, and lifestyle modifications.

## 2. Migraine Prevalence

**Figure 1:**
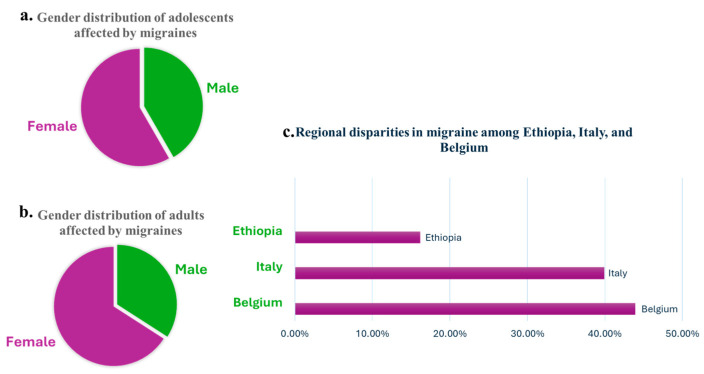
Overview of migraine prevalence and distribution: (**a**) gender distribution of adolescents affected by migraines, showing a higher prevalence among girls compared to boys; (**b**) gender distribution of adults affected by migraines, emphasizing a greater proportion of women compared to men; (**c**) comparison of migraine prevalence in selected high-income (Belgium and Italy) and low-income (Ethiopia) countries, used as examples to highlight disparities in healthcare access and diagnostic capabilities.

Migraine is a highly prevalent neurological disorder, affecting approximately 1.1 billion people globally as of 2019, and ranks as the second leading cause of years lived with disability (YLDs), contributing to significant societal and individual burden [3]. Chronic migraine affects 1–2% of the population, and 2.5% of individuals with episodic migraines will develop chronic migraine over time [14]. Migraine has significant demographic variations, as its prevalence is notably higher in women, reaching nearly 18.9% compared to 9.8% in men (Figure 1), largely due to hormonal influences such as estrogen fluctuations [15]. It affects approximately 5% of youth by age 10 and increases in prevalence throughout adolescence, with nearly 28% of girls and 20% of boys experiencing migraines by their late teenage years (Figure 1) [1,16]. While regional disparities in prevalence exist, high-income countries like Belgium and Italy report the highest rates (22,400 and 20,337 per 100,000) and lower rates are reported in regions like Ethiopia (8277 per 100,000), highlighting differences in healthcare access and diagnostic capabilities (Figure 1) [15,16]. Additionally, despite a decline in active migraines among older populations, up to 25.7% of women aged 60 and older continue to live with inactive migraines, underscoring the long-term impact of the condition [16]. These statistics emphasize the urgent need for innovative, accessible, and holistic interventions that address the multifaceted burden of migraine and improve quality of life for diverse populations and age groups worldwide.

## 3. Migraine Phases

**Figure 2:**
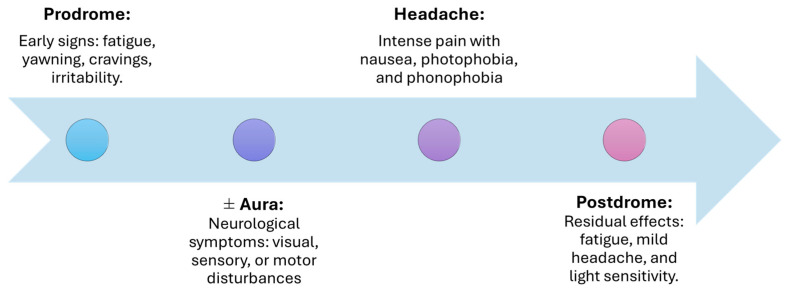
Phases of migraine: from onset to resolution.

A comprehensive understanding of this progression, which unfolds in four phases: prodromal, aura, headache, and postdromal (Figure 2), is crucial for elucidating both the variability of the migraine experience and the underlying pathophysiological mechanisms.

### 3.1. Prodromal Phase

The period of subclinical signs and symptoms that precedes the onset of a migraine headache is referred to as the prodrome phase. This phase occurs 24–48 h before headaches and is marked by early symptoms such as fatigue, yawning, irritability, muscle stiffness, food cravings, nausea, and sleeping difficulty. These symptoms suggest early dysfunction in the hypothalamus and brainstem, which prime the nervous system for the subsequent phases [17,18].

### 3.2. Aura Phase

Aura refers to a range of neurological symptoms—visual, sensory, speech, auditory, and motor disturbances—that can occur before or during a migraine attack. Experienced by about 25% of migraine sufferers, these symptoms include transient issues like visual flashes, changes in hearing, or motor disruptions. Caused by cortical spreading depression, these symptoms typically develop over 5 to 60 min and are fully reversible [18,19].

### 3.3. Headache Phase

The hallmark of migraine, the headache phase, can last from several hours to three days. Pain is typically unilateral but may become bilateral and is often accompanied by nausea, photophobia, and phonophobia. The activation of the trigeminovascular system and the release of neuropeptides like CGRP drive the pain and associated symptoms during this phase [17,18].

### 3.4. Postdromal Phase

The postdromal phase, also called the “migraine hangover”, occurs after the headache subsides. Lingering fatigue, mild headache, and sensitivity to light or smells are common. This phase reflects the residual activation of pain pathways and can be aggravated by environmental triggers [17,18].

## 4. Migraine Pathophysiology

The intricate biology of migraine involves multifactorial mechanisms that disrupt central and peripheral sensory processing, resulting in episodic or chronic pain and associated symptoms. Understanding these mechanisms is essential to appreciate the disorder’s complexity and guide effective treatment strategies.

### 4.1. Trigeminovascular System

Migraine pain primarily stems from the activation of the trigeminovascular system. This system involves nerve fibers from the trigeminal ganglion, which connect to the blood vessels in the cranial meninges. When triggered, these fibers release neuropeptides, such as CGRP. CGRP acts as a strong vasodilator, causing blood vessels to expand and trigger inflammation. This process heightens the sensitivity of pain pathways in the brain, which sustains the migraine [9,20,21,22]. By understanding how this system links both vascular and neural processes, it is clear why therapies targeting CGRP, such as CGRP antagonists, are an effective approach to migraine treatment.

### 4.2. Cortical Spreading Depression (CSD)

CSD is a self-propagating wave of neuronal depolarization and hyperpolarization strongly associated with aura symptoms. It activates the trigeminovascular system and releases inflammatory mediators, contributing to the initiation of headache. While pivotal in the aura phase, CSD is considered an epiphenomenon in migraine without aura [23,24].

### 4.3. Brainstem and Hypothalamic Dysfunction

The brainstem, particularly the periaqueductal gray (PAG) and locus coeruleus, modulates pain perception and plays a vital role during migraine episodes. Concurrently, the hypothalamus regulates premonitory symptoms such as yawning, thirst, and fatigue, reflecting its influence on cyclic migraine patterns. Functional imaging reveals altered connectivity between the hypothalamus, brainstem, and cortical areas in migraine patients, underscoring their role in initiating and perpetuating migraine attacks [25].

### 4.4. Neuroinflammation and Sensitization

Neurogenic inflammation amplifies migraine pain. During attacks, pro-inflammatory mediators like CGRP and substance P are released, leading to vasodilation, mast cell degranulation, and increased nitric oxide production. These processes sensitize trigeminal afferents, escalating pain and causing symptoms like photophobia and allodynia [22,24].

### 4.5. Thalamic and Cortical Involvement

The thalamus and cortex are central to migraine pathophysiology, contributing to sensory, cognitive, and emotional symptoms. The thalamus acts as a relay, amplifying nociceptive signals from the trigeminovascular system, which heightens pain perception and sensory sensitivities like photophobia and phonophobia. It also integrates signals from other brain regions, influencing the emotional and cognitive aspects of migraines. Structural and functional changes in cortical regions, particularly the occipital cortex, are linked to aura and visual symptoms, while dysregulated cortical networks further enhance pain perception [25].

## 5. Migraine-Contributing Risk Factors

Migraine episodes are deeply influenced by a variety of triggers, making their identification and management vital for reducing attack frequency and severity. Among the most significant contributors, stress stands out, affecting over 70% of patients and highlighting the profound connection between emotional strain and migraines [26,27]. Hormonal fluctuations, particularly during menstruation, are another powerful trigger, increasing migraine risk by up to 96%, underscoring the need for gender-specific approaches [28,29]. Dietary factors like fasting, alcohol, and chocolate frequently precipitate attacks, while environmental triggers such as noise, strong odors, and weather change further complicate its management [26,28,29]. Interestingly, some factors, like relaxation after stress, may unexpectedly reduce migraine risk, emphasizing the complexity of these triggers [28]. To provide a comprehensive understanding, these influences are categorized into intrinsic factors, such as hormonal changes and genetic predisposition, and extrinsic factors, including environmental and lifestyle elements, with each discussed in detail.

### 5.1. Intrinsic Risk Factors

Intrinsic factors originate within the individual and are closely linked to their biological and physiological makeup.

#### 5.1.1. Genetic and Epigenetic Factors

Genetics plays a crucial role in determining susceptibility to migraines, with specific mutations strongly linked to certain migraine subtypes. Familial hemiplegic migraine (FHM), a rare and severe form, is inherited in an autosomal dominant pattern, meaning that a single copy of the mutated gene from either parent is enough to increase the risk of developing the condition. This pattern of inheritance means that if one parent carries the mutation, each child has a 50% chance of inheriting it [30]. FHM is associated with mutations in the CACNA1A, ATP1A2, and SCN1A genes, which affect ion channels responsible for regulating neuronal signaling. These mutations lead to an increased level of neuronal excitability, which can trigger migraine episodes, along with symptoms like temporary motor weakness and heightened sensitivity to light or sound [31]. Additionally, genetic factors influencing glutamate neurotransmission, synaptic development, and ion channel homeostasis contribute to migraine risk [18]. A large meta-analysis involving 375,000 individuals identified several migraine-related single nucleotide polymorphisms linked to arterial and smooth muscle function, suggesting a connection between vascular health and migraine pathogenesis. Furthermore, epigenetic changes, such as DNA methylation, can alter gene expression related to pain and inflammation, further elevating migraine susceptibility [32].

#### 5.1.2. Hormonal Imbalances

Fluctuations in hormone levels, particularly estrogen, significantly affect migraines, explaining their higher prevalence in women [33]. Estrogen influences pain-processing pathways in the brain by modulating neurotransmitters like serotonin and dopamine. Drops in estrogen levels, such as those occurring before menstruation or during menopause, can destabilize these pathways, leading to heightened sensitivity to pain and triggering migraine episodes [34,35].

Similarly, elevated cortisol levels during stress disrupt circadian rhythms, which regulate essential functions like sleep–wake cycles. Chronic cortisol elevation increases brain excitability, heightening sensitivity to pain and triggering migraine attacks [36]. Additionally, circadian disruptions lead to poor sleep, a well-known migraine trigger. Together, these factors amplify the frequency and severity of migraines, highlighting the importance of stress management in prevention [37,38].

#### 5.1.3. Metabolic Factors

Metabolic disorders, including obesity, diabetes, hypertension, and dyslipidemia, contribute to vascular changes and systemic inflammation, which are critical in migraine pathophysiology. For example, obesity is linked to increased inflammatory markers and altered adipokine levels (signaling proteins secreted by fat tissue that regulate inflammation and metabolism), both of which can amplify neuronal excitability [39]. Hypertension and dyslipidemia can compromise blood flow regulation in the brain, triggering migraines or prolonging their duration [40,41]. Interestingly, while type 1 diabetes may have a protective effect, type 2 diabetes shows no clear association, highlighting complex interplays between metabolic health and migraines [37,42].

#### 5.1.4. Neurological Disorders

Neurological conditions, such as epilepsy and multiple sclerosis (MS), frequently co-occur with migraines due to shared pathways. Both epilepsy and migraines involve paroxysmal episodes caused by neuronal hyperexcitability, with evidence suggesting overlapping genetic and biochemical mechanisms [43]. Similarly, in MS, inflammation and demyelination in the central nervous system can disrupt neural communication, leading to migraines as a secondary manifestation [44]. Movement disorders like restless leg syndrome and Parkinson’s disease also share dopaminergic and iron metabolism dysfunctions, which may explain their link to migraines [37,45,46,47].

#### 5.1.5. Psychological and Psychiatric Factors

Psychological stress and psychiatric conditions are among the most potent contributors to migraines. Depression and anxiety disorders are particularly common, with stress acting as both a trigger and a consequence of migraines. Chronic stress can sensitize pain pathways in the brain, lowering the threshold for migraine attacks [37,48,49]. Similarly, conditions like post-traumatic stress disorder and bipolar disorder involve disruptions in neurotransmitters like serotonin and dopamine, which also regulate pain perception [50]. This bidirectional relationship between psychiatric health and migraines underscores the importance of holistic management strategies.

### 5.2. Extrinsic Risk Factors

Extrinsic factors are external influences that interact with the individual’s internal environment to precipitate or exacerbate migraines.

#### 5.2.1. Lifestyle and Behavioral Triggers

Disruptions in sleep patterns, such as insomnia or obstructive sleep apnea, are strongly linked to migraines. Sleep deprivation impairs the brain’s ability to regulate pain by increasing cortical excitability and inflammatory responses. Dietary habits, including fasting or irregular meal patterns, can destabilize glucose levels, triggering migraines. Additionally, excessive alcohol consumption is a well-known trigger, though the mechanism remains unclear; it likely involves the dilation of blood vessels and inflammatory responses [51,52].

#### 5.2.2. Environmental Factors

Environmental stressors such as noise, strong odors, and bright or flickering lights can act as direct triggers for migraines by overstimulating sensory pathways. Changes in weather, including barometric pressure drops, have been shown to precipitate migraines in sensitive individuals, likely due to vascular responses in the brain. Prolonged exposure to stressful environments, whether social or occupational, not only increases the frequency of migraines but also contributes to their chronicity by maintaining heightened pain sensitivity [37,53,54].

#### 5.2.3. Medication Overuse

The overuse of acute migraine medications, such as triptans, opioids, or analgesics, paradoxically increases migraine frequency and can lead to medication overuse headaches (MOHs) [55]. This occurs through mechanisms like central sensitization, where the brain becomes increasingly sensitive to pain signals, and the disruption of pain modulation pathways, which exacerbates dependency and diminishes the efficacy of treatments [56]. Over time, this misuse induces neuroinflammatory and neurotoxic changes in critical pain-processing regions, such as the trigeminal nucleus caudalis and the periaqueductal gray matter, while the withdrawal–rebound mechanism perpetuates a harmful cycle of escalating medication use [57]. Additionally, failure to effectively manage early migraine episodes can contribute to the progression from episodic to chronic migraines, with structural changes observed in pain-processing circuits [55,56,57]. Addressing medication overuse is vital to breaking this cycle and preventing long-term consequences, emphasizing the importance of preventive therapies, multidisciplinary care, and patient education tailored to reduce reliance on acute medications and promote sustainable headache management.

## 6. Search Strategy

To conduct a comprehensive literature review on the efficacy of natural therapies and complementary approaches for migraine management, we searched academic databases including PubMed, Google Scholar, EKB, and PsycINFO. We used MeSH terms and keywords such as “migraine”, “natural products”, “herbal remedies”, “aromatherapy”, “dietary supplements”, “lifestyle modifications”, and “alternative medicine”. We combined keywords with Boolean operators (AND and OR) for precision, e.g., “migraine AND natural products” or “herbal remedies OR clinical studies”. We focused on studies published since 2010 to prioritize recent advancements, emphasizing reviews and observational studies that assess the efficacy of natural supplements for managing migraines. We excluded non-English studies, case reports, editorials, and those with unclear methodologies or results to maintain relevance and rigor.

## 7. Results

### 7.1. Acute and Preventive Medications for Migraine Management

Migraine management involves both acute and preventive therapies tailored to individual patient needs. Acute treatments aim to relieve symptoms during attacks, while preventive therapies reduce the frequency, intensity, and duration of migraines over time. Medications for acute relief include triptans, NSAIDs, and gepants, and they are selected based on migraine severity, patient-specific factors, and prior treatment responses. Preventive options, such as beta-blockers, anticonvulsants, and CGRP monoclonal antibodies, are intended for patients with frequent or severe migraines that significantly impair quality of life [58,59]. The following tables (Table 1 and Table 2) summarize the mechanisms, efficacy, side effects, and safety considerations for these medications.

The complexity and variability of migraines, combined with the limitations of current pharmacological treatments, highlight the need for alternative and complementary strategies. While medications such as triptans, beta-blockers, and CGRP inhibitors are effective, they often carry challenges such as side effects, including cardiovascular risks, medication overuse headaches (MOHs), and adherence issues. These limitations underscore the importance of developing holistic approaches that integrate natural therapies with evidence-based preventive treatments to address the diverse needs of migraine patients.

Natural and complementary therapies have gained attention as potential alternatives or adjuncts to conventional migraine treatments. Feverfew has shown promise in reducing migraine frequency, yet several studies have failed to replicate these outcomes, likely due to differences in trial design, dosing, and product standardization [86]. Butterbur, despite its effectiveness, is overshadowed by safety concerns, including hepatotoxicity, necessitating the use of pyrrolizidine alkaloid-free formulations to mitigate risks [87]. Ginger is primarily recognized for its acute effects, such as alleviating nausea and vomiting, but its role in prevention remains inconclusive due to mixed clinical evidence [88,89]. Other natural therapies, including dietary supplements like magnesium and riboflavin, show potential in both preventive and acute settings [90,91]. However, these approaches require rigorous clinical validation to confirm their efficacy and safety.

When compared to conventional treatments, natural therapies are generally more accessible and affordable, particularly in resource-constrained settings. For example, feverfew and magnesium supplements are widely available over the counter, reducing barriers to access. However, their inconsistent clinical outcomes and limited regulatory oversight raise concerns about their efficacy and safety [92,93]. In contrast, conventional treatments, such as CGRP inhibitors and beta-blockers, are backed by robust clinical evidence and regulatory approval, offering more predictable outcomes. CGRP inhibitors, including Erenumab and Fremanezumab, have demonstrated significant efficacy in reducing migraine frequency, particularly in patients unresponsive to older therapies, but their high costs and limited availability in low-income regions present substantial barriers to access [94]. Preventive therapies, such as beta-blockers and anticonvulsants, are effective, but they are often associated with side effects like fatigue, cognitive dysfunction, and mood disturbances which can lead to adherence challenges [8,71,72,73,74,75,76,77,78,79,80].

To address these gaps, combining evidence-based natural products with validated preventive treatments like CGRP inhibitors could optimize outcomes by reducing reliance on acute medications and enhancing cost-effectiveness. Rigorous clinical validation and the standardization of natural therapies are also needed to ensure safety and efficacy, paving the way for their integration into evidence-based migraine management.

### 7.2. Herbal Products (Plant Extracts, Pure Compounds, or Their Supplements) for Migraine Management

Tracing the literature highlights the pivotal role of natural products in migraine treatment. Today, several herbal supplements containing plant extracts or purified plant constituents are available for both prophylactic and abortive migraine management. Randomized clinical trials, along with in vivo, in vitro, and molecular docking studies, have demonstrated the effectiveness and safety of numerous natural products. These include plants like feverfew, butterbur, ginger, and ginkgo, as well as phytoconstituents such as parthenolide, petasin, isopetasin, curcumin, ergotamine, dihydroergotamine, Δ9-tetrahydrocannabinol, and cannabidiol.

**Figure 3:**
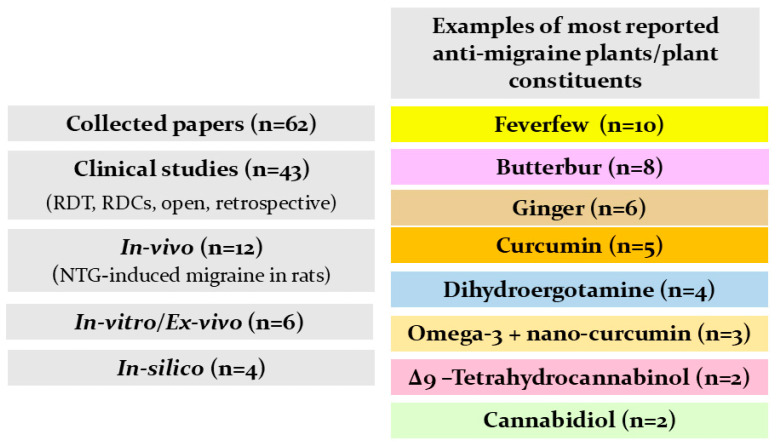
Literature searching scheme for natural plants and plant constituents used for migraine management.

**Figure 4:**
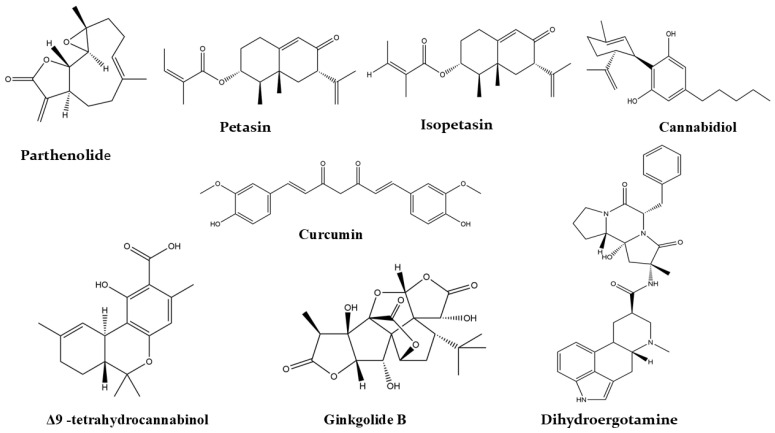
Selected chemical structures of plant constituents for the management of migraine.

**Figure 5:**
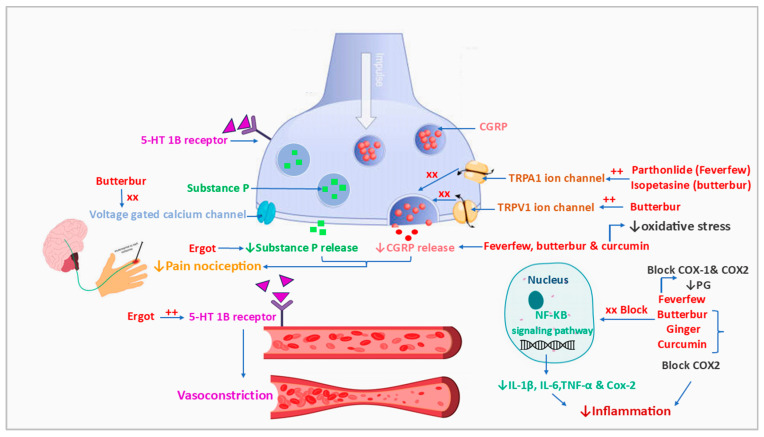
Mechanisms of action of natural products or phytochemicals involved in migraine treatment. CGRP; calcitonin gene-related peptide, Cox-1; cyclooxiginase-1 enzyme, Cox-2; cyclooxiginase-2 enzyme, 5-HT-1B; 5-hydroxytryptamin 1B (serotonin receptor), IL; interleukin, NF-KB; nuclear factor kappa-light-chain-enhancer of activated B cells, PG; prostaglandin, TNF-α; tumor necrosis factor alpha, TRPA1; transient receptor potential ankyrin 1, TRPV1; transient receptor potential cation channel subfamily V member 1, also known as vanilloid receptor 1 and capsaicin receptor.

Figure 3 summarizes our findings from the literature on natural products studied for their anti-migraine activity. Reported investigations into herbal supplements, Ayurvedic formulations, plant extracts, and isolated plant constituents with demonstrated efficacy are presented in Table 3. Additionally, the chemical structures of key plant-derived constituents with anti-migraine activity are depicted in Figure 4. The mechanisms of action of natural products or phytochemicals involved in migraine treatment were displayed in Figure 5.

#### 7.2.1. Feverfew

Feverfew (*Tanacetum parthenium* (L.) Sch. Bip.), a member of the Asteraceae family, has traditionally been used to treat fever, asthma, and inflammatory conditions such as psoriasis, insect bites, and rheumatism [95]. Modern research highlights its efficacy in migraine prophylaxis, and this is attributed primarily to parthenolide, a sesquiterpene lactone that inhibits serotonin release and prostaglandin biosynthesis, key mechanisms in migraine pathophysiology [86]. Furthermore, feverfew exhibits antiplatelet activity by suppressing thromboxane synthesis, which may address the vascular components of migraines [96].

Standardized supercritical CO_2_ extracts of feverfew, either alone or combined with other herbs such as *Salix alba* L. (white willow) or ginger, are commonly incorporated into migraine-specific herbal supplements. These formulations, designed for migraine management, include MIG-99 and Mig-RL (capsules), GelStat Migraine (sublingual), and LipiGesic and Antemig (tablets) [97,98,99,100]. While feverfew generally causes mild side effects, including nausea, stomach upset, and skin irritation, it is contraindicated in pregnancy due to its emmenagogue properties, in children under two years, and in individuals that are allergic to Asteraceae plants [96,101].

#### 7.2.2. Butterbur

Butterbur (*Petasites hybridus* (L.) G. Gaertn., B. Mey. and Scherb.), another Asteraceae family member, has been traditionally used for central nervous system conditions, including migraine, hypertension, and dysmenorrhea [87]. Studies have demonstrated that its anti-migraine effects are primarily due to its active sesquiterpenes, petasin, and isopetasin. These compounds exert their effects through multiple mechanisms, including blocking voltage-dependent calcium channels, inhibiting CGRP secretion, and activating TRPA1 and TRPV1 channels. These actions help desensitize nociceptors and reduce inflammation, key factors in migraine pathophysiology [102,103,104,105]. Randomized clinical trials confirm the efficacy of standardized butterbur extracts (containing up to 15% petasin) in migraine prophylaxis for adults, adolescents, and children [106,107,108]. Products like Petadolex have shown promise but must be pyrrolizidine alkaloid-free to avoid hepatotoxicity [87].

#### 7.2.3. Ginger

Ginger *(Zingiber officinale* Roscoe), a member of the Zingiberaceae family and a staple in Ayurvedic medicine, is traditionally used for digestive and respiratory ailments [109]. Randomized clinical trials have shown that ginger rhizome powder or extracts (400–500 mg/day, standardized to ≥5% gingerol) are effective for acute migraine relief, reducing pain intensity and duration within two hours [109,110,111,112]. Notably, combined formulations like GelStat and LipiGesic, which pair ginger with feverfew, show enhanced efficacy [98].

Ginger’s anti-migraine activity is primarily attributed to bioactive compounds like gingerols and shogaols, which inhibit cyclooxygenase-2, thereby reducing prostaglandin biosynthesis. Additionally, ginger’s antiemetic properties mitigate nausea and vomiting commonly associated with migraines [88]. Despite its benefits in acute management, studies investigating prophylactic effects have yielded mixed results, suggesting that ginger’s role may be limited to symptomatic relief [89].

#### 7.2.4. Curcumin

Curcumin, a polyphenol from turmeric (*Curcuma longa* L.), exhibits antioxidant and anti-inflammatory properties, making it a potential migraine therapy. Its poor bioavailability necessitates advanced formulations, such as nanoparticles and liposomes, to enhance its absorption [113]. Clinical trials have highlighted curcumin’s benefits in reducing pain severity, oxidative stress, and inflammatory mediators like CGRP and IL-6. For instance, 100 mg/day of curcumin or 250 mg/day of phytosomal curcumin over two months has shown symptom improvement [114,115]. Synergistic effects have been observed when combined with omega-3 fatty acids or fish oil [116,117,118]. Preclinical studies in animal models have further demonstrated its antioxidant and analgesic effects, especially when paired with other drugs like sumatriptan or naproxen [119,120,121].

**Table 3:** Reported herbal supplements or their constituents for migraine.

Plant/Herbal Supplement	Plant Part	Extract Type/ Composition	Study Design	Method	Result	References
**Preclinical Data**						
Butterbur	Root	Isopetasin (isolated from the acetone extract)	In vitro In vivo	The ability of isopetasin to target TRPA1 channel was tested in vitro in rodent and human TRPA1-expressing cells using patch-clamp recording and single-cell calcium imaging. CGRP release was tested in mouse spinal cord. Meningeal blood flow and facial rubbing were tested in vivo in rats and mice.	Isopetasin could activate TRPA1 channels, causing the excitation of neuropeptide containing nociceptors and the desensitization of peptidergic trigeminal nerve terminals, attenuating their ability to release CGRP.	[105]
Butterbur	-	Petasin Isopetasin (isolated from the ethanolic extract)	In vitro	The mechanism of anti-migraine activity of petasin and isopetasin was tested in vitro by evaluating CGRP release in the hemisected skull of a mouse and rats and dissected trigeminal ganglia.	Petasin and isopetasin are the active constituents of butterbur root extract; they reduced CGRP levels by affecting both TRPA1 and TRPV1 receptor channels.	[104]
-	-	Curcumin	In vivo	NTG-induced rats treated with either curcumin (10 mg/100 g), propranolol (100 µg/100 g), or Indomethacin (0.5 mg/100 g). The control group was treated with NTG only, and the non-migraine group was treated with normal saline. Formalin test and oxidative stress parameters were determined.	Curcumin showed an analgesic effect, reduced nociception (↓ number of shakes and flinches) when received before pain stimuli, and enhanced antioxidant activity (↓ NO, TOS, and MDA)	[119]
-	-	Curcumin Liposomal curcumin	In vivo	NTG-treated rats were divided into 6 groups and received either NTG only (control group), sumatriptan only (1 mg/100 g), 2 different doses of curcumin (1 mg/100 g and 2 mg/100 g) along with sumatriptan, or 2 different doses of liposomal curcumin along with sumatriptan. In addition, the non-migraine control group received normal saline. Formalin test and oxidative parameters were measured	Liposomal curcumin (2 mg/100 g) adjuvant with sumatriptan showed the most powerful potential for reducing nociception (↓ number of shakes and flinches), and it improved antioxidant potential (↑ TAC levels and serum thiol). ↓ NO, TOS, and MDA in all curcumin groups, especially liposomal curcumin (2 mg/100 g)	[120]
-	-	Curcumin Liposomal curcumin	In vivo	NTG-treated rats were divided into 6 groups and received either NTG only (control group), naproxen only (2.8 mg/kg), 2 different doses of curcumin (1 mg/100 g and 2 mg/100 g) along with naproxen, or 2 different doses of liposomal curcumin along with naproxen. In addition, the non-migraine control group received normal saline. Formalin test and oxidative parameters were measured	Combination of liposomal curcumin with naproxen showed enhanced anti-nociception and antioxidant potential. (↑ TAC levels and ↓ NO, TOS, and MDA in all curcumin-treated groups)	[121]
-	-	Cannabidiol	In vivo	Cannabidiol anti-migraine activity was tested in a CGRP-induced migraine mice model. Cephalic allodynia, anxiety-like behavior, spontaneous pain, and photophobia were determined	Cannabidiol could be used as an abortive treatment for migraine attacks. It reversed CGRP-evoked allodynia, decreased pain traits in female mice, and relieved anxiety in male mice.	[122]
-	-	Δ9-tetrahydrocannabinol (THC)	In vivo	The anti-migraine potential of THC was tested in rats using AITC-induced depression of wheel running. Rats were injected with 10 µL of 10% AITC or mineral oil in the dura mater, then immediately injected with either THC or vehicle at different doses: 0.1, 0.32, and 1 mg/kg	THC was effective in treating migraine-like pain when taken immediately after AITC at a dose of 0.32 mg/kg. The anti-migraine potential is related to CB1.	[123]
Feverfew	Flowering arial parts	Parthenolide isolated from the ethanolic extract	In vitro In vivo	Electrophysiologic recording, calcium imaging, and CGRP-like immunoreactivity assays were performed in vitro using human- and mouse-cultured neurons, cells, and tissues isolated from rats. Changes in meningeal blood flow and the eye wiping assay were used to detect nociceptive response; behavior testing and dural cannulation were evaluated in in vivo models using mice and rats.	Parthenolide may produce the anti-migraine activity by acting as a partial agonist to TRPA1, desensitizing the TRPA1 channel to any stimulus, which resulted in the inhibition of CGRP release.	[124]
Feverfew	-	Methanol combined with hexane, dichloromethane, and ether (standardized to (0.5% parthenolide *w*/*w*)	In vivo	Rats in treatment groups received either 100 mg/kg of FE or 100 mg/kg of FD and 15 mg/kg of purified parthenolide for 6 days followed by NTG injection, and they were compared with 3 groups that received the same medication without NTG. Control groups received either vehicle or NTG. The effect of extracts and purified parthenolide on NTG-induced Fos expression was measured as a marker for neural activation	Feverfew extracts enriched with parthenolide showed reduced Fos expression in the nucleus trigeminalis caudalis. Purified parthenolide inhibited neuronal activation in another brain nuclei and nuclear factor-kB.	[125]
Feverfew *Salix alba*	-	Water extract (standardized to (0.5% parthenolide *w*/*w*) and 15% salicin *w*/*w*)	Ex vivo In silico	The effect of combined feverfew and Salix alba extracts on serotonin, nitrites, and lactate dehydrogenase levels were determined in cortex tissue homogenates of mice via HPLC. A docking study of major detected metabolites; the caffeic acid and epicatechin to MAO-A enzyme assay was performed.	The combination of feverfew and Salix alba extracts showed a protective effect against migraines by reducing lactate, nitrites, and serotonin degradation. The prevention of serotonin depletion may be due to the inhibition of MAO-A enzyme that is responsible for serotonin catabolism using caffeic acid and epicatechin	[126]
*Ficus deltoidea*	Leaves	A standardized aqueous extract was obtained from Zach Biotech Sdn. Bhd, Malaysia.	In vivo	The anti-hyperalgesic behavior of *F. deltoidea* leaves’ aqueous extract (50, 100, and 200 mg/kg daily) and C-fos expression level in TNC were evaluated in NTG-induced mice.	*F. deltoidea* at 100 and 200 mg/kg showed a marked reduction in the nociceptive behaviors induced by NTG and inhibited C-Fos production.	[127]
Formula of Ayurvedic herbs (consisted of 8 plants, 10 g each)	Fruits of *Terminalia bellerica*, *Terminalia chebula*, *Piper nigrum*, and *Emblica officinalis*. Roots of *Valerina villaichi* and *Premna Integrifolia*. *Tinospora cordifolia* stem and *Azadiracta indica* bark	Hydro–alcoholic extract (50:50); yield is standardized to 6–8 gm.	In vivo	The migraine activity of the extract was assessed in mice using 3 models; morphine withdrawal caused hyperalgesia in the hot plate test; acetic acid-induced abdominal constrictions; and apomorphine induced climbing.	The Ayurvedic herb extract showed promising efficacy for anti-migraine activity by acting on dopamine.	[128]
*Ginkgo biloba* EGb 761^®^	Leaves	Standardized extract	In vivo	Rats were divided into 6 groups. The control, model, and sham operation groups received distilled water, and 3 groups received Gingko biloba extract at different doses of 25, 50, and 100 mg/kg for 1 week. Then, NTGs were injected subcutaneously into the gingko biloba and sham operation groups.	*Gingo biloba* could regulate nitric oxide, platelets aggregation rate, and 5-hydroxytryptamine levels during migraine attacks.	[129]
*Ligusticum chuanxiong* Hort. (LCH)	Edible herb	Aqueous extract	In vivo	The migraine activity of LCH aqueous extract was tested in NTG-induced rats. Cerebral blood flow was detected using laser speckle contrast imaging. Molecular changes were determined via immunofluorescence, RT-qPCR, and immunohistochemistry.	LCH could effectively reduce cerebral injury and migraine pain attack threshold by regulating glutamate, glucose metabolism, and Nrf-2 signals	[130]
*Sapindus trifoliatus* L.	Pericarp	Aqueous extract	In vitro	The aqueous extract of *S. trifoliatus* was tested in vitro to illustrate its action to the following receptors; HT1B/1D, 5-HT2B, GABA, Ca^2+^ channel, vanilloid receptor, and platelet serotonin release assay.	*Sapindus trifoliatus* showed a prophylactic effect against migraine via the mediation of the 5-HT2B receptor and the inhibition of serotonin release from platelets	[131]
Yufeng Ningxin tablet (YNT)	*Pueraria lobata* root	-	In vivo In vitro In silico	An in silico study using network pharmacology analysis. An in vivo model of NTG-induced rats was used to evaluate 3 doses of YNT (378, 576, and 1512 mg/kg, orally) for 10 days. Cortex and serum CGRP, 5-HT, IL-1β, NF-κB, and c-fos were determined. YNT extract was tested in vitro on LPS-induced NF-κB p65 expression in BV2 cells.	A network pharmacology analysis showed that the major anti-migraine targets for YNT constituents were 5-HT, NF-κB, CGRP, nociceptive factors, and inflammation. The in vivo study revealed that YNT could elevate 5-HT expression and decrease NF-κB, CGRP, IL-1β, and c-fos. YNT inhibited LPS-induced inflammation through NF-κB in BV2 cells.	[132]
Clinical data						
Antemig^®^, PiLeJe tablets	-	Feverfew Coenzyme Q10 Magnesium	Clinical study	Patients received 1 tablet/day consisting of 100 mg of feverfew, 100 mg of coenzyme Q10, and 112.5 mg of magnesium, as well as 1.4 mg of vitamin B6 for 3 months; after 1 month, baseline period without treatment was established.	The consumption of Antemig^®^ supplement at 1 tablet/day for 3 months exerted a prophylactic effect against migraine by reducing the number of attacks over the period of use.	[133]
Butterbur	Root	Extract standardized to at least 15% petasins	RCT	Patients (n = 33) received butterbur root extract at 50 mg/twice daily or placebo (n = 27) for 3 months.	Butterbur extract showed an effective role in migraine prophylaxis. The frequency of attacks reduced by 52.9% after 3 months of use.	[134]
Butterbur	Root	Extract standardized to at least 15% petasins	RCT	Patients (n = 245) were divided into 3 groups and received either butterbur extract 50 mg/twice daily, 75 mg/twice daily, or placebo for 4 months.	Butterbur extract at 75 mg/twice daily could reduce the frequency of migraine attacks in 68% of patients after 4 months of treatment.	[107]
Butterbur	Root	Extract standardized to at least 15% petasins	RCT	Patients (n = 108), children and adolescents (age 6–17 years), received 50 to 150 mg of butterbur root extract according to their age for 4 months.	Butterbur root extract showed an effective prophylactic effect against migraine in children and adolescents. A total of 77% of the patients showed a reduced number of migraine attacks by 50%.	[108]
Butterbur	Root	Extract standardized to at least 15% petasins	RCT	Patients (n = 58) were divided into 3 groups; each group received either butterbur root extract, music therapy, or placebo for 3 months. The frequency of attacks was assessed at 2- and 6-month post treatment.	The music group showed a reduction in the number of migraine attacks post treatment, while after 6 months, both the music therapy and butterbur root extract groups showed a prophylactic effect.	[135]
Curcuma	-	Curcumin supplements contained a standardized 95% turmeric extract in pellet form (475 mg curcuminoids, covering 70–80% curcumin, 15–20% dimethoxy curcumin and 2.5–6.5% bis-demethoxy curcumin)	RCT	Women patients (n = 44) with migraine received either 500 mg of curcumin twice/day or placebo for 2 months. Serum CGRP and IL-6, as well as pain severity, duration, and frequency were measured.	Curcumin showed a significant decrease in CGRP and IL-6, as well as in pain severity and duration.	[136]
-	-	Nano-curcumin	RCT	Patients diagnosed with episodic migraine (n = 40) were randomly assigned to receive 80 mg/day of nano-curcumin or placebo for 2 months. Gene expression and serum levels of TGF-b and IL-4 in PBMCs isolated from the migraine patients were determined via real-time PCR and ELISA, respectively.	The nano-curcumin intake showed group a significant increase in serum levels of IL-4, exerting anti-inflammatory effects, while no remarkable action was seen for serum levels and gene expression of TGF-b.	[137]
-	-	Phytosomal curcumin	RCT	Patients (n = 60) with migraine received either 250 mg/day phytosomal curcumin or maltodextrin as placebo for 2 months. NO, hs-CRP, and oxidative stress were measured	The intake of phytosomal curcumin at 250 mg/day for 2 months could relief symptoms of migraine and inhibit oxidative stress and inflammation.	[115]
-	-	Omega-3 fatty acids Nano-curcumin	RCT	Patients (n = 72) with episodic migraine were randomized into 4 groups: group 1 received 2 capsules/day of omega-3 (600 mg DHA, 1200 mg EPA and 200 mg other omega-3); group 2 received 1 capsule containing 80 mg of nanocurcumin/day; group 3 took 2 capsules of omega-3 and 1 capsule of nanocurcumin/day; group 4 received placebo. The gene expression and serum level of ICAM-1 were measured after 2 months of consumption.	The combination of omega-3 and nanocurcumin for 2 months showed a significant decrease in frequency of migraine attacks and serum level of ICAM-1, while no significant difference was seen in the gene expression of ICAM-1.	[116]
-	-	Omega-3 fatty acids Nano-curcumin	RCT	Patients (n = 80) with episodic migraine were randomized into 4 groups: group 1 received 2 capsules/day of omega-3 (600 mg DHA,1200 mg EPA and 200 mg other omega-3); group 2 received 1 capsule containing 80 mg of nanocurcumin/day; group 3 took 2 capsules of omega-3 and 1 capsule of nanocurcumin/day; group 4; received placebo. The gene expression and serum level of VCAM were measured after 2 months of consumption.	The combination of omega-3 and nanocurcumin for 2 months showed a significant decrease in gene expression and serum level of VCAM compared to the intake of omega-3 alone. No significant change was seen in patients who received nanocurcumin alone.	[118]
-	-	Omega-3 fatty acids Nano-curcumin	RCT	Patients with episodic migraine (n = 80) were randomized into 4 groups: group 1: 2500 mg/day of fish oil capsule and 80 mg/day of nano-curcumin; group 2: 2500 mg/day of fish oil capsule; group 3: 80 mg/day of nano-curcumin; group 4: paraffin oil placebo capsule. The serum level and gene expression of IL-1β were determined after 2 months of treatment.	The combination of omega-3 fatty acids and nano-curcumin showed a significant decrease in the frequency of migraine attacks and serum level of IL-1β, while a non-significant differences was seen in the gene expression of IL-1β.	[117]
-	-	Δ9-tetrahydrocannabinol (THC) Cannabidiol (CBD)	RCT	Patients (n = 92) were divided into 4 groups and received, for 4 separate acute migraine attacks, either vaporized 6% THC, 11% CBD; a combination of 6% THC + 11% CBD; and cannabis flower as placebo.	The combination of 6% THC and 11% CBD showed an effective relief of acute migraine pain after 2 h of intake and a sustained effect for 24 and 48 h.	[138]
Feverfew	Arial parts	80% Ethanolic extract (standardized to 0.4% parthenolide)	RCT	Patients (n = 60) were divided into 2 groups. Both groups received 40 mg/day of propranolol in the form of a tablet and 10 drops of parthenolide (0.4%) or placebo for 3 months. The MIDAS questionnaire was used to evaluate the results.	Feverfew extract exerted a prophylactic effect against migraine. The frequency and severity of migraine attacks decreased by 61.62% and 56.2%, respectively, for 3 months.	[139]
-	-	Magnesium at 300 mg (1:1 ratio of magnesium citrate and oxide), riboflavin at 400 mg, and feverfew at 100 mg (standardized to 0.7% parthenolide)	RCT	Patients received a combination of feverfew at 100 mg, riboflavin at 400 mg, and magnesium at 300 mg for 3 months, while placebo group took 25 mg riboflavin daily.	A combination of feverfew, riboflavin, and magnesium showed a similar effect to the placebo.	[140]
GelStat Migraine^®^	-	Feverfew Ginger	Clinical study	Patients (n = 30) received 2 mL of feverfew/ginger sublingually when they felt mild pain	GelStat Migraine^®^ could relieve mild pain after 2 h in 48% of the patients.	[99]
Ginger	-	A total of 500 mg/d of ginger (two capsules of 250 mg Zintoma (Gol Darou Co.) along with 500 mg/d of Depakene.	RDBCT	Patients (n = 80) were randomized into 2 groups. The treatment group received 2 ginger capsules (500 mg/day) orally and Depakene (500 mg/day of sodium valproate) for 4 months, while the control group received only Depakene at 500 mg/day.	Ginger intake at 500 mg/day adjunct to Depakene could relief the pain intensity of migraine compared to using Depakene alone.	[112]
Ginger	Rhizome	Standardized to 5% gingerols.	RCT	Patients (n = 103) were randomization into 2 groups. The treatment group received 500 mg/day ginger dry extract, and the control group received placebo starch for 3 months.	The ginger dry extract group showed reduced duration and severity of migraine attacks compared to placebo. No significant effect on the number of migraine attacks.	[111]
Ginger	-	Extract standardized to at least 5% gingerols	RCT	Participants with acute migraine were randomized into 2 groups that received 400 mg of ginger extract or placebo, and both received 100 mg of ketoprofen.	Ginger could be used as an adjuvant to non-steroidal anti-inflammatory drugs to relief acute migraine pain.	[110]
Ginger	-	Extract standardized to at least 5% gingerols	RCT	Patients (n = 107) with chronic migraine were randomized into treatment and placebo groups that received capsules (200 mg, 3 times/day) of ginger dry extract or placebo (cellulose) for 3 months.	Ginger showed no significant effect on migraine prophylaxis compared to the placebo.	[89]
Ginger	Rhizome	Extract standardized to at least 5% gingerols.	RDBCT	Patients (n = 100) with acute migraine without aura were randomly divided into 2 groups and received either 1 capsule containing 250 mg/day of ginger powder or 50 mg sumatriptan for 1 month.	The activity of ginger powder was similar to sumatriptan in relieving the pain of acute migraine without aura within 2 h of use.	[109]
Iaraj Fiqra tablet	Rose flower, cinnamon bark, ginger rhizome, *Aloe vera*, saffron flower, balsam incense, asaroon root, *Pistacia lentiscus* fruit, *Commiphora opobalsamum* balsam	-	RCT	Patients received a 500 mg/day Iaraj Fiqra tablet for 2 months. Headache was tested using the MIDAS HIT-6 test.	Iaraj Fiqra herbal supplements showed a significant decrease in duration, frequency, and severity of migraine attacks compared to placebo	[141]
Jodeungsan (JDS) (Granular product of herbal supplement consisted of 11 plants)	Uncariae Ramulus et Uncus Liriopis seu Ophiopogonis tuber, Pinelliae tuber, *Poria Sclerotium*, Ginseng radix, *Saposhnikoviae radix*, *Citri unshius* pericarp, *Chrysanthemi Indici* flower, Zingiberis rhizome, *Glycyrrhizae Radix* et rhizome, *Gypsum Fibrosum*	-	RCT	Patients (n = 32 at each group) were given 22.5 g/day of JDS or placebo for 28 days.	JDS showed no significant difference on chronic migraine in HAD compared to the control group. However, the MSQoL and HIT results exerted significant enhancements in both groups.	[142]
-	-	Flaxseed oil L-carnitine	RCT	Women patients with migraine were assigned into 2 groups (n = 40, each); placebo group and treatment group that received flaxseed oil (1000 mg/day) and L-carnitine (500 mg/day) for 3 months	Flaxseed oil and L-carnitine intake showed a marked reduction in migraine symptoms, oxidative stress, inflammation, and enhanced quality of life.	[143]
LipiGesic^TM^M	-	Feverfew Ginger	RCT	Patients (n = 45) took sublingual feverfew/ginger for 163 migraine attacks for 1 month.	The feverfew/ginger combination showed a relief of mild pain before the onset of severe or moderate pain after 2 h in 63% of the patients.	[98]
Migrasoll^®^	-	*Ginkgo biloba* terpenes phytosome (Ginkgolide B) Coenzyme Q 10 Vitamin B2	Clinical study	Patients (n = 50) with migraine with aura received 120 mg of ginkgo biloba terpenes phytosome, 17.4 mg of vitamin B2, and 22 mg of coenzyme Q 10 daily for 4 months.	Ginkgolide B terpenes was effective in decreasing the duration and frequency of migraine attacks with aura after two months of use.	[144]
Migrasoll^®^	-	*Ginkgo biloba* terpenes phytosome (Ginkgolide B) Coenzyme Q 10 Vitamin B2	Clinical study	Patients (n = 25) diagnosed with migraine with aura received 120 mg of ginkgo biloba terpenes phytosome, 17.4 mg of vitamin B2, and 22 mg of coenzyme Q 10 at the onset of second attack.	Ginkgolide B, the terpene fraction of ginkgo, showed a significant decrease in duration of neurological symptoms in 60% of the patients of acute migraine with aura, and the pain disappeared completely in 18% of the patients.	[145]
-	-	Ginkgolide B Riboflavin Coenzyme Q10 Magnesium	Clinical study	Children patients (n = 119; mean age 9.7 ± 1.42) suffering from migraine without aura received a combination of ginkgolide B, riboflavin, coenzyme Q10, and magnesium for 3 months	The combination of ginkgolide B, riboflavin, coenzyme Q10, and magnesium was effective as a prophylaxis treatment of childhood migraine without aura by reducing the frequency of attacks.	[146]
-	-	Ginkgolide B Coenzyme Q 10 Vitamin B2 Magnesium	Clinical study	Young patients (n = 30, age: 8–18) suffering from migraine without aura received 160 mg of ginkgolide B, 3.2 mg of vitamin B2, 40 mg of coenzyme Q, and 400 mg of magnesium daily for 3 months.	Ginkgolide B showed a prophylactic effect against migraine without aura in young patients. It could decrease the number of attacks and the regular use of analgesics.	[147]
MIG-99 (Soft gelatin capsule)	Feverfew (Flowering arial parts)	Supercritical CO_2_ extraction	RDBCT	The patients (n = 147) were divided into 3 groups, and each took a different dose of feverfew extract: 2.08 mg, 6.25 mg, and 18.75 mg 3 times/daily for 12 weeks. The efficacy of the treatment during the last 4 weeks were compared to the 4-week baseline period before starting the treatment.	MIG-99 did not show a significant prophylactic effect. Feverfew extract (6.25 mg 3 times/daily) could reduce the number of migraine attacks in a small group of patients that received at least 4 attacks/month.	[97]
MIG-99	Feverfew (Flowering arial parts)	Supercritical CO_2_ extraction	RCT	Patients (n = 170) were divided into 2 groups and received either 18.75 mg of feverfew extract or placebo for 4 months after 1 month at baseline.	Feverfew was effective in the prophylaxis of migraine using 18.75 mg/day for 4 months. It could reduce the number of attacks by 1.9/month compared to baseline.	[148]
MYRLIQ^®^	-	*Ginkgo biloba* L. Vitamin B2 Coenzyme Q10	Clinical pilot study	Patients suffering from migraine headache without aura or tension-type headache received a combination of Ginkgo biloba, vitamin B2, and coenzyme Q10 at the start of headache attack for 6 months.	The natural combination of Ginkgo biloba, vitamin B2, and coenzyme Q10 showed analgesic and anti-inflammatory activity. It could relief the pain in 75% of the patients and reduce inflammatory markers such as TNF-α, IL6, and IL-8.	[149]
Neurasites^®^ (A standardized extract of butterbur)	-	Butterbur (Petasites hybridus) 150 mg contains at least 7.5% petasin	Clinical Study	Migraine activity was assessed through the Neurasites^®^ Questionnaire Survey (NQS) in 85 patients assigned in 2 groups; placebo and Neurasites^®^-based treatment, using imigran as positive controls for migraine with aura and topiramate for chronic migraine.	Neurasites^®^ could effectively reduce the intensity of pain, the duration of headache attacks, and the numbers of migraine episodes.	[150]
Petadolex^®^	Rhizome	CO_2_ extract, standardized to at least 15% petasins.	RDBCT	Patients (n = 60) received 2 Petadolex capsules (each containing 25 mg of butterbur extract) twice daily or placebo for 3 months.	Butterbur showed a prophylactic effect against migraine by reducing the number of migraine attacks by 60%	[106]
Computational data						
-	-	Asarinin	In silico	Molecular docking of asarinin with CGRP receptor, molecular dynamic assessment, MMGBSA analysis, and network analysis were used to compared the anti-migraine drug Atogepant	Asarinin was considered a promising anti migraine candidate. It showed good binding affinity (−10.3 kcal/mol) with CGRP receptor (PDB: 6ZHO). Network analysis highlighted the major proteins that interacted with the targets of migraine with aura such as OPRM1, GNB1, and GNAS	[151]
Ginger	-	98% Ethanol	In silico	The anti-migraine activity of ginger constituents detected via GC-MS was evaluated using molecular docking and compared to the anti-migraine drug Telcagepant.	Among 28 tested compounds; Estra-1,3,5(10)-trien-17-beta-ol showed the best binding scores (6.8 and −8.8 1 Kcalmol^−1^) in CGRP (PDB:3n7r) and human NK1 tachykinin receptors (PDB:6e59), respectively, which are very close to the control Telcagepant	[152]

AITC; allyl isothiocyanate, CB1; cannabinoid receptor 1, CGRP; calcitonin gene-related peptide, FD; feverfew-depleted extract of parthenolide, feverfew-enriched parthenolide, GNAS; gene protein subunit alpha S, GNB1; gene protein subunit beta 1, HAD; headache attack days, HIT; headache impact test, HPLC; high-performance liquid chromatography, hs-CRP; high-sensitivity C-reactive protein, 5-HT; 5-hydroxytryptamine, ICAM-1; intercellular adhesion molecule-1, IL; interleukin, LPS; lipopolysaccharides, MAO-A; monoamineoxxidase-A, MDA; malondialdehyde, MIDAS; migraine disability assessment, MSQoL; migraine-specific quality of life, NF-κB; nuclear factor kappa, Nrf-2; nuclear factor erythroid 2-related factor 2, NO; nitric oxide, NTG; nitroglycerin, OPRM1; opioid receptor Mu 1, PBMCs; peripheral blood mononuclear cells, RDBCT; randomized double-blind clinical trial, RCT; randomized double-blind, placebo-controlled clinical study, TGF-b; Transforming growth factor beta, TNF-*α*; tumor necrosis factor alpha, TOS; total oxidative capacity, TRPA1; transient receptor potential ankyrin 1, TRPV1; transient receptor potential cation channel subfamily V member 1, known as vanilloid receptor 1 and capsaicin receptor, TNC; trigeminal nucleus caudalis, VCAM; vascular cell adhesion molecule.

### 7.3. Complementary (Non-Pharmacological) Approaches

**Figure 6:**
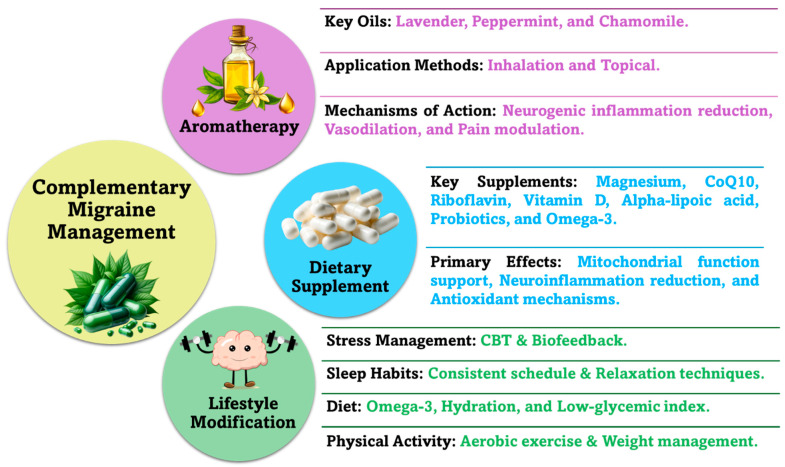
Complementary (non-pharmacological) approaches in migraine management.

Aromatherapy, dietary supplements, and lifestyle modifications are increasingly recognized as effective complementary treatments for migraines, as illustrated in Figure 6.

#### 7.3.1. Migraine and Aromatherapy

Aromatherapy, the use of high-quality plant-based essential oils through inhalation or topical application, is increasingly recognized as a complementary approach for managing migraines [153,154]. This practice offers an alternative to conventional pharmacological treatments, which are often accompanied by side effects and contraindications [155]. Essential oils contain bioactive compounds such as terpenoids, esters, and aldehydes, which contribute to their therapeutic effects [156]. Specifically, research has demonstrated that essential oils can alleviate migraine symptoms such as nausea, photophobia, and phonophobia by modulating neurotransmitter levels, reducing neurogenic inflammation, and promoting vascular relaxation. For instance, lavender oil’s topical application to the temples has been associated with reflex actions that relax blood vessels and reduce pain perception, highlighting its unique mechanism of action [155].

The method of essential oil application plays a crucial role in optimizing therapeutic effects. Topical application, such as to the temples or forehead, utilizes the oils’ skin penetration properties, while inhalation leverages their volatility to activate sensory pathways. The choice of application method depends on the oil’s properties and the patient’s tolerance to optimize therapeutic effects [154]. For example, gentle oils like lavender are often applied directly to the skin, while oils such as peppermint are usually diluted in a carrier oil to appropriate concentrations to prevent skin irritation. Additionally, some essential oils are formulated into suitable pharmaceutical forms, like gels, for targeted application. Inhalation methods can accommodate flexible concentrations, ranging from 1% to 100%, depending on the enclosed space, making them adaptable to individual needs [155].

Several essential oils were evaluated for their effectiveness in managing migraines. Essential oils such as lavender, peppermint, chamomile, anise, basil, rose, and a mixed blend of lavender and grape seed oil demonstrated promising outcomes as essential oils aromatherapy in reducing migraine intensity, frequency, and symptoms like nausea, photophobia, and phonophobia. These oils primarily work through mechanisms targeting vasodilation, the inhibition of neurogenic inflammation, and a reduction in central pain sensitization, offering multifaceted relief in both experimental and clinical settings [157].

##### Lavender Oil

Lavender oil, one of the most extensively studied essential oils in aromatherapy, has shown significant effectiveness in anti-migraine clinical and animal studies. It mainly contains linalool and linalyl acetate as its main active constituents. Clinical trials involved patients applying 2–3 drops of lavender oil to their upper lips for inhalation at the onset of migraine symptoms, while animal models used inhalation at concentrations of 1%, 10%, and 100% in controlled environments. Both human and animal studies found significant reductions in headache severity, nausea, and sensitivity to light and sound within two hours of inhalation. Lavender’s active components, such as linalool and linalyl acetate, contribute to its effects by relaxing blood vessels, inhibiting neurogenic inflammation, and potentially modulating neurotransmitter activity. This combination of actions makes lavender oil a valuable option for managing acute migraine symptoms [155,158].

##### Peppermint Oil

Peppermint oil has also been widely researched for its anti-migraine properties. In clinical settings, peppermint oil diluted to 1.5% was applied to the nostrils during the onset of migraines. Patients experienced relief within five minutes, with notable reductions in both headache intensity and frequency like the lidocaine group [159]. The rapid action of peppermint oil is largely attributed to menthol, a key compound that activates TRPM8 receptors, producing a cooling sensation that helps modulate pain and inflammation. Additionally, peppermint oil promotes vasodilation, making it highly effective in managing acute migraine symptoms [160].

##### Basil Oil

Basil oil has been studied in clinical trials where it was applied topically on the forehead and temples in concentrations of 2%, 4%, and 6%. Over the study period, patients experienced gradual declines in migraine intensity and attack frequency. The oil’s active components, such as estragole and eugenol, are thought to contribute to basil’s therapeutic effects by modulating blood flow and reducing vascular tension. Through a combination of vasodilation and anti-inflammatory actions, basil oil offers both immediate and preventive migraine relief [161].

##### Chamomile Oil

Chamomile oil has been examined for its potential to relieve migraine symptoms when applied topically. In clinical trials, a 5.5% chamomile essential oil gel was applied to the temples, forehead, and areas behind the ears. Within 30 min, patients reported a decrease in pain intensity and relief from associated symptoms such as nausea, photophobia, and phonophobia. The oil’s active components, including chamazulene and bisabolol, are known for their anti-inflammatory properties, probably helping to inhibit neurogenic inflammation and reduce pain sensitization. Chamomile oil’s capacity to alleviate a broad range of migraine symptoms makes it especially useful for holistic migraine management [162].

##### Anise Oil

Anise oil, used in aromatherapy, has also shown promise in clinical trials, where a 7% anise essential oil cream was applied to the temples and forehead during migraine episodes. Routine application led to a reduction in migraine frequency from an average of 3.45 episodes per week to 1.89, and it decreased the average duration of migraines by approximately twelve hours [163]. The active compound anethole has notable anti-inflammatory properties, targeting the neurogenic inflammation associated with migraine pain. Anethole is structural similarity to dopamine, potentially allowing it to act as a dopamine receptor antagonist, thereby interrupting the dopamine-related cascade that induces migraines [164]. Also, anise oil’s preventive effects make it valuable for those experiencing frequent migraines.

##### Rose Oil

Rose oil, extracted from *Rosa damascena*, has shown particular effectiveness in managing “hot” migraines, which are characterized by symptoms like facial heat and red eyes. Clinical studies involved applying rose oil to the forehead and temples with a formulation derived from soaking rose petals in 20% sesame oil. This treatment significantly reduced pain intensity in patients with hot migraine syndrome. Rose oil’s active compounds, citronellol and geraniol, are believed to calm vascular responses and reduce inflammation, making it well suited for migraines with pronounced vasodilation-related symptoms [165].

##### Lavender Oil and Grape Seed Oil

A mixed essential oil blend of lavender and grape seed oil has shown promising results for migraine relief in aromatherapy. In clinical settings, this blend, formulated with a 3:2:10 ratio of lavender to grape seed to base oil, was applied to the forehead, face, and neck. Over a 20-day period, most patients reported complete symptom relief, with some experiencing longer intervals between migraine attacks. This combination is effective due to the synergy of lavender’s anti-inflammatory and vasodilatory properties with grape seed oil’s antioxidant effects, providing comprehensive relief from both migraine pain and its associated symptoms [155,157].

#### 7.3.2. Migraine and Dietary Supplements

Dietary supplements are increasingly recognized as an effective complementary approach to managing migraines, much like aromatherapy. Oxidative stress and low antioxidant intake are significant risk factors for migraines, as they can trigger inflammation and neuroinflammatory responses. Research shows that supplements targeting mitochondrial function, neuroinflammation, brain energy metabolism, and vascular health can help reduce migraine frequency and severity. Nutrients such as Coenzyme Q10, riboflavin, omega-3 fatty acids, magnesium, alpha-lipoic acid, and probiotics have shown promising effects by addressing deficiencies or physiological imbalances linked to migraine pathogenesis, helping to stabilize cellular energy, reduce neuroinflammation, and regulate pathways involved in migraine attacks [166].

##### Magnesium

Magnesium, the fourth most abundant cation in the body, is crucial for nerve function, enzyme activity, DNA and protein synthesis, and maintaining the electrical potential of neurons. It is found mainly in leafy green vegetables as well as in nuts and seeds. Deficiency in magnesium, due to inadequate intake, excessive loss, or cellular redistribution, is a risk factor for migraines, potentially due to its impact on neurotransmission and vascular health [166]. Magnesium deficiency has been linked to migraine pathogenesis through mechanisms such as platelet aggregation, glutamate release, and decreased mitochondrial function, which promote the formation of vasoconstrictive agents like 5-hydroxytryptamine (5-HT), and they are often associated with migraine onset [167,168]. A U.S. National Health and Nutrition Study (including data from 3626 participants) and research by Assarzadegan et al. both reported significantly lower serum magnesium levels in individuals with migraines compared to healthy controls [169,170]. Clinical studies also highlight the effectiveness of magnesium supplementation in migraine management. Chiu et al. found that intravenous magnesium quickly alleviated acute migraine symptoms, while oral magnesium reduced attack frequency and intensity [90]. Moreover, Khani et al. reported that combining magnesium with sodium valproate was more effective than either treatment alone, lowering attack frequency, intensity, and medication requirements [171]. Given the prevalence of magnesium deficiency in migraine sufferers, magnesium supplementation, especially as adjunct therapy, holds potential for enhancing migraine treatment outcomes.

##### Coenzyme Q10

Coenzyme Q10 (CoQ10) is a natural antioxidant crucial for mitochondrial energy production and cellular respiration functioning within the electron transport chain. Known for its benefits in conditions like cardiovascular disease, fibromyalgia, and neurodegenerative disorders, CoQ10 may also help prevent migraines [166]. Migraine has been associated with mitochondrial dysfunction, specifically energy deficits affecting oxidative phosphorylation, which can disrupt vascular tone and promote oxidative stress. CoQ10 supports mitochondrial function, reduces inflammation, and has shown negative associations with inflammatory markers, which may influence migraine pathogenesis [172]. Several studies highlight CoQ10’s potential for migraine management. A clinical trial demonstrated that 400 mg/day of CoQ10 for three months reduced headache severity, frequency, and duration, as well as inflammatory markers like tumor necrosis factor-α (TNF-α) and calcitonin gene-related peptide (CGRP) [173]. Shoeibi et al. found that 100 mg/day of CoQ10 reduced migraine attack severity and frequency [174], while other trials noted improvements in oxidative stress markers and HDL cholesterol in migraine patients receiving 400 mg/day of CoQ10 [175]. A meta-analysis of six randomized trials confirmed CoQ10’s efficacy in lowering attack frequency, with enhanced effects when used as adjunct therapy [176]. Although evidence supports CoQ10’s safety and efficacy in reducing migraine symptoms, further studies are recommended to confirm its optimal dosing and effects in combination therapies.

##### Riboflavin

Riboflavin (vitamin B2) is a water-soluble vitamin that converts vitamins into the cofactors flavin mononucleotide (FMN) and flavin–adenosine dinucleotide (FAD), both essential for flavoenzymes in the electron transport chain, which is vital for cellular energy production [91]. Riboflavin also has anti-inflammatory, antioxidant, anti-nociceptive, and anti-aging properties [166]. It is primarily found in dairy products and green vegetables, although its structure is not susceptible to cooking heat, and it can be degraded by light. Riboflavin deficiency, affecting about 10–15% of the population due to genetic issues, is associated with neuroinflammation and conditions like migraines [177,178]. Several studies have demonstrated the effectiveness of riboflavin supplementation in preventing migraines. High doses (400 mg/day) have shown reductions in attack frequency, duration, severity, and the use of anti-migraine medications [91]. Even low doses (100 mg/day) were found to be as effective as propranolol, a common migraine treatment [179]. Additionally, riboflavin’s effects were comparable to sodium valproate (500 mg/day) for migraine prophylaxis [180]. Based on these findings, riboflavin is considered a promising option for migraine prevention, with supplementation for at least 12 weeks being beneficial [179]. Further research is needed to determine the optimal dosage and duration.

##### Vitamin D

Vitamin D, a fat-soluble vitamin primarily synthesized in the skin through sunlight exposure and obtained from dietary sources like fatty fish, fortified foods, and supplements, is crucial for calcium absorption, bone health, and various cellular processes. Its active form, calcitriol, binds to nuclear vitamin D receptors (nVDRs) in the central nervous system, modulating neuroplasticity, oxidative stress, calcium homeostasis, immune responses, and neurotrophic factor production [166]. Vitamin D deficiency, affecting 30–80% of the global population, has been linked to neurodegenerative and neurological conditions, including migraines [166,181]. Studies demonstrate a connection between vitamin D deficiency and increased migraine risk. Rapisarda et al. found that migraine patients often exhibit severe vitamin D deficiency, with an inverse relationship between serum vitamin D levels and headache frequency [182]. Clinical trials, including those by Mottaghi et al. and Ghorbani et al., reveal that vitamin D supplementation (e.g., 50,000 IU weekly or 2000 IU daily) significantly reduces migraine frequency, severity, and associated inflammatory markers like IL-6 and iNOS [183,184]. Hu et al.’s meta-analysis confirms vitamin D’s efficacy in lowering headache days and disability scores, though its effects on attack duration and intensity remain inconsistent [185]. While promising, further studies are needed to solidify vitamin D’s role in migraine management and establish optimal dosing regimens.

##### Alpha-Lipoic Acid

Alpha-lipoic acid (α-LA) is an organosulfur antioxidant synthesized in the mitochondria, and it is essential in energy metabolism and antioxidant defense. Acting as a cofactor in enzyme complexes, α-LA supports mitochondrial function and has high potential in neutralizing ROS and RNS, which are elevated in migraines due to oxidative stress and mitochondrial dysfunction. This imbalance, involving damaged cellular components such as DNA, proteins, and lipids, is a key factor in migraine pathogenesis [166]. Several studies highlight α-LA’s potential in migraine management. Eren et al. found a significant correlation between low thiol levels and migraine severity, suggesting that thiol-containing compounds like α-LA may offer therapeutic benefits [186]. Gross et al. reported abnormally low serum α-LA levels in migraine patients, indicating it as a potential biomarker for prevention and treatment [187]. Clinical trials support these findings. Rezaei et al. observed that 300 mg/day of α-LA for three months reduced markers of oxidative stress and inflammation, including MDA and CRP in women [188]. Cavestro et al. found that 400 mg/day of α-LA over six months reduced headache days in migraine patients with insulin resistance [189], while Magis et al. showed that 600 mg/day over three months reduced migraine frequency and severity [190]. Despite the positive results, further studies are needed to confirm optimal dosing and its mechanisms, though current evidence suggests α-LA’s safety and efficacy in reducing oxidative stress and improving symptoms associated with migraine.

##### Probiotics

Probiotics, often derived from fermented foods or supplements, are live microorganisms that support health by enhancing gut microbiota balance. Psychobiotics, a class of probiotics, specifically benefit brain function by affecting neuronal pathways through the gut–brain axis. Migraine, a neurovascular condition often linked to gut dysbiosis, appears to be influenced by interactions between the gut and the brain via neural, immune, and endocrine pathways. Research highlights that gut microbiota imbalance can contribute to inflammation and increase intestinal permeability, allowing pro-inflammatory substances to enter the bloodstream and activate trigeminal pathways, potentially triggering migraine attacks. Stress-induced changes in the hypothalamic–pituitary–adrenal axis, which elevate cortisol and weaken gut barrier integrity, are additional factors [166]. Martami et al. found that daily probiotic supplements reduced migraine frequency, severity, and medication use in both episodic and chronic migraine patients, although attack duration decreased only in chronic cases [191]. Studies by Xie et al. and Ghavami et al. also demonstrated that probiotics, especially when paired with an elimination diet or combined with prebiotics, decreased migraine attacks, medication use, and inflammation markers like zonulin and Hs-CRP [192,193]. However, inconsistent results across studies, likely due to varying probiotic formulations and microbial strains, highlight the need for further large-scale trials to confirm probiotics’ role in migraine management.

##### Omega-3

Omega-3 polyunsaturated fatty acids (PUFAs), such as eicosapentaenoic acid and docosahexaenoic acid, play an important role in managing migraine. Using rich sources of these omega-3s like fish oil increases the PUFA content, which exhibit anti-inflammatory and neuroprotective mechanisms. These fatty acids reduce neuroinflammation by suppressing cytokine production, inhibiting NF-κB activity via lipopolysaccharide (LPS) receptors, and decreasing the production of NO and ROS in active microglia. They also influence transcription factors, intracellular signaling pathways, and gene expression, contributing to their antinociceptive effects [166]. Clinical studies support these mechanisms, and a recent randomized controlled trial (RCT) demonstrated that supplementing a diet with 1.8 g/day of eicosapentaenoic acid over 12 weeks reduced attack severity, frequency, and associated disability while improving quality of life [194]. Another study found that combining 180 mg/day of fish oil with sodium valproate was more effective than the medication alone in reducing attack frequency [195]. Diets rich in omega-3 and low in omega-6 further enhanced pain relief and antinociceptive lipid mediator levels [196]. Although omega-3 supplementation has shown benefits in reducing migraine attack frequency and improving quality of life, further research is needed to determine optimal dosages and treatment protocols.

#### 7.3.3. Migraine and Lifestyle

**Figure 7:**
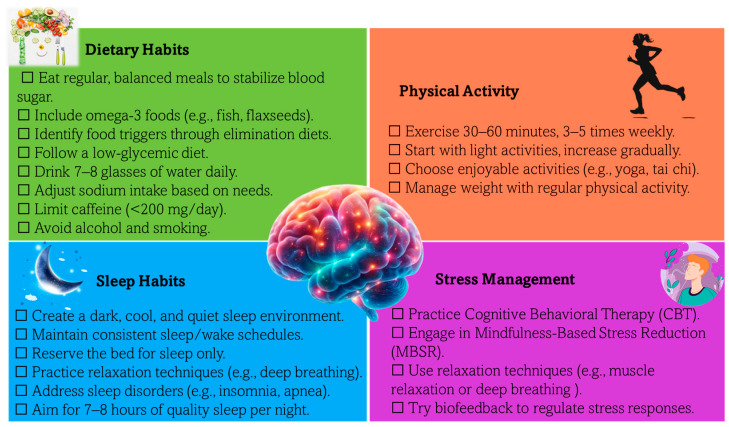
Checklist of lifestyle modifications for effective migraine management. This visual summary highlights evidence-based lifestyle changes, including dietary, sleep, physical activity, and stress management strategies, aimed at reducing migraine frequency, severity, and overall burden while enhancing quality of life.

Migraine imposes a significant burden on patients, affecting daily functioning, productivity, and overall well-being. While pharmacological treatment is essential, lifestyle modifications have emerged as critical, non-pharmacological strategies to reduce migraine frequency and severity. These changes target modifiable risk factors such as the overuse of acute migraine medications, metabolic syndrome, obesity, depression, and stress. [51]. Figure 7 summarizes evidence-based lifestyle strategies, including dietary habits, physical activity, sleep optimization, and stress management, which have been shown to alleviate migraine symptoms and enhance quality of life.

##### Dietary Habits

Balanced meals, omega-3 fatty acids, hydration, and caffeine management play a pivotal role in stabilizing blood sugar and preventing triggers. Elimination diets and low-glycemic food choices offer personalized approaches to migraine prevention [51,52]. Specific details on dietary recommendations are illustrated in Figure 7.

##### Sleep Optimization

Maintaining a consistent sleep schedule, optimizing the sleep environment, and addressing disorders like insomnia or apnea are essential for migraine management. Aim for 7–8 h of quality sleep per night to reduce attack frequency and severity [51,52].

##### Physical Activity

Regular exercise, such as aerobic activity or yoga, not only helps reduce migraine frequency and intensity but also supports weight management, a key factor in mitigating risk.

##### Stress Management

Techniques such as cognitive behavioral therapy (CBT), mindfulness-based stress reduction (MBSR), relaxation techniques, and biofeedback are proven to decrease migraine-related anxiety and stress [51,52,197,198,199,200].

Together, these modifications represent sustainable, low-cost approaches to migraine management. For a detailed, actionable checklist of recommendations, refer to Figure 7, which provides an easy-to-follow guide for patients and practitioners alike.

## 8. Future Prospectives

Embracing a personalized approach by tailoring natural supplement regimens to individual patient profiles and incorporating genetic predispositions, lifestyle factors, and comorbidities, holds the key to optimizing treatment efficacy and outcomes in migraine management. To substantiate the effectiveness and safety of natural supplements, further research through well-designed clinical trials and longitudinal studies is imperative. This robust evidence will not only guide clinical decision-making but also elevate the standard of patient care. By exploring synergies among natural supplements, prescription medications, behavioral therapies, and other non-pharmacological interventions, a novel and comprehensive treatment landscape can be unveiled for migraine sufferers. Empowering patients with comprehensive knowledge about natural supplements, their mechanisms of action, and potential benefits is crucial for informed decision-making and active involvement in their treatment plans. Collaborative efforts among diverse healthcare providers, including neurologists, primary care physicians, and complementary medicine practitioners, are essential to establish a holistic and integrated approach to migraine care, ensuring comprehensive support for patients on their healing journey. Establishing clear guidelines and standards for the quality, purity, and labeling of natural supplements will further bolster consumer safety and confidence in their utilization as adjunct therapies for migraine relief. By embracing these forward-looking perspectives and nurturing interdisciplinary collaboration, the field of migraine management is poised for a transformative evolution, offering innovative and patient-centered strategies that effectively address the intricate nature of migraines while enhancing overall care quality and patient outcomes.

## 9. Conclusions

By adopting a broader, more integrative perspective than systematic reviews, this review bridges traditional pharmacological insights with emerging evidence on natural therapies, aiming to deepen the understanding of migraine management, guide clinical practice and future research, and empower individuals to manage their condition more effectively. While prescription medications remain cornerstone interventions, the integration of natural supplements offers a personalized and comprehensive strategy that may address gaps in current treatment paradigms. Non-pharmacological approaches, such as lifestyle modifications and dietary habits, are increasingly recognized as critical components of migraine management.

## Figures and Tables

**Table 1:** Acute migraine relief medications.

Medication Class	Examples	Mechanism of Action	Efficacy	Side Effects	Safety Concerns	References
Triptans	Sumatriptan, Rizatriptan	Selective serotonin (5-HT1B/1D) receptor agonists; induce vasoconstriction and inhibit nociceptive transmission.	Effective in ~70% of patients; relief within 2 h; best when taken at the prodrome stage.	Chest tightness, flushing, dizziness.	Contraindicated in cardiovascular diseases; risk of serotonin syndrome with SSRIs/SNRIs.	[58,60,61,62]
NSAIDs	Ibuprofen, Naproxen	Inhibit COX enzymes to reduce prostaglandin synthesis and inflammation.	Effective for mild to moderate migraines; comparable to triptans in some cases.	GI upset, nausea, risk of MOH.	Avoid peptic ulcers, renal dysfunction, or cardiovascular risk, long-term use risks GI bleeding.	[63,64,65,66]
Gepants	Rimegepant, Ubrogepant	CGRP receptor antagonists; block vasodilation and neurogenic inflammation.	Effective in triptan-refractory patients; relief within 2 h; low recurrence risk.	Nausea, dry mouth, somnolence.	Monitor for hepatic/renal impairment; adjust dose with CYP3A4-interacting drugs.	[67,68,69,70]

**Table 2:** Preventive migraine medications.

Medication Class	Examples	Mechanism of Action	Efficacy	Side Effects	Safety Concerns	References
Beta-Blockers	Propranolol, Metoprolol	Reduce neuronal excitability and stabilize vascular tone.	Reduce frequency by ~50% in ~50% of patients, especially with hypertension.	Fatigue, bradycardia, sleep disturbances.	Contraindicated in asthma/COPD and bradycardia; require gradual titration.	[71,72,73,74]
Anticonvulsants	Topiramate, Valproate	Modulate neurotransmitters and reduce cortical spreading depression.	Reduce frequency by ~50%; effective in comorbid mood disorders.	Cognitive dysfunction, weight changes, tremor; valproate risks hepatotoxicity, teratogenicity.	Avoid valproate in pregnancy; monitor liver and metabolic function for both.	[8,75,76,77,78,79,80]
CGRP Monoclonal Antibodies	Erenumab, Fremanezumab	Block CGRP or its receptor to prevent central and peripheral sensitization.		Injection site reactions, constipation, muscle spasms.	Limited long-term safety data; monitor for possible immunogenic effects.	[81,82,83,84,85]

## Data Availability

All data are available within the manuscript.
